# 

**Published:** 1889-05-25

**Authors:** 


SATURDAY, MAY 25th, 1889.

**Catalepsy or Death** - - - - - - - - 111

**Turning Over New Leaves.**—For the Right - - - 112

**The Doctor's Pulpit.**—Man and his Enemies - - - 113

**The Causation of Disease.**—II. - - - - - - 114

**The Hospital Workshop.**—With the Sick Children, Great Ormond-street - - - - - - - - - 115

**Editor's Letter-Box.**—Football Brutalities - - - 116

**The Bishop of London's Fund.**—Bishop Temple reports progress and asks for further help - - - 117

**Within the Wards.**—Hysterical Men - - - - 117

**Notes and News** - - - - - - - - 118

**Everybody's Page** - - - - - - - - - 120

**Annotations.**—Cabby's Progress—Frogs or Beefsteaks—A Plea for Football - - - - - - - - 121

**The National Pension Fund for Nurses** - - - 122

**"Her Heart's Mistake."** By E. D. Cumming, Author of "An Ordeal by Silence" - - - - - - 123

**Heredity** - - - - - - - - - - 125

**Amusements and Relaxation** - - - - - 126

**Scraps and Gleanings** - - - - - - 126

EXTRA SUPPLEMENT—THE NURSING MIRROR.

**En Passant.**—Examination Questions—Short Items—Royalty and Nursing—Wigan Infirmary—Nurses at Belfast - - - - - - - - - xxvii
**The British Nurses Association and the Press** xxviii
**Nursing in the Soudan.**—The End of the War xxviii
**A Nurse's Diary.**—The First Week in Hospital - - xxix
**Everybody's Opinion.**—The Age of Nurses—Major Mears Scheme—Almshouses - - - - - - - xxx
**The Nurse's Bookshelf** - - - - - - xxx
**Lectures.—Examination Questions** - - - - xxx
**Vacancies** - - - - - - - - - - xxx
**Notes and Queries** - - - - - - - - xxx

